# Supplementary material for: Polyketide synthase gene clusters in the ericoid mycorrhizal fungus Oidiodendron maius: genome-wide prediction, expression profiling, and functional characterization of knockout mutants
Source: Front Plant Sci. 2026 Sep 16;17:1924430. doi: 10.3389/fpls.2026.1924430 (PMC13623903; doi:10.3389/fpls.2026.1924430)
Supplement: Supplementary file 1 [file SupplementaryFile1.zip › Supplementary Materials/Supplementary Folder 1.zip!/Oidma1_AssemblyScaffolds_Repeatmasked/knownclusterblast/cluster45/jgi.p_Oidma1_60475_mibig_hits.html]

| MIBiG Protein | Description | MIBiG Cluster | MiBiG Product | % ID | % Coverage | BLAST Score | E-value |
| --- | --- | --- | --- | --- | --- | --- | --- |
| AAO62580.1 | phosphoglycerate\_dehydrogenase-like\_protein | BGC0001016 | Microcystin\_biosynthetic\_gene\_cluster | 27 | 69.0410958904 | 80 | 4.1e-15 |
| AAF00955.1 | McyI | BGC0001017 | Microcystin\_biosynthetic\_gene\_cluster | 31 | 45.7534246575 | 66 | 1e-10 |
